# Supplementary material for: Integrative discovery of treatments for high-risk neuroblastoma
Source: Nat Commun. 2020 Jan 3;11:71. doi: 10.1038/s41467-019-13817-8 (PMC6941971; doi:10.1038/s41467-019-13817-8)
Supplement: Supplementary file 1 — Supplementary Information [file 41467_2019_13817_MOESM1_ESM.pdf]

Integrative discovery of treatments for high-risk neuroblastoma

Almstedt et al.

Supplementary Methods

TargetTranslator method. TargetTranslator has three main steps: 1) Combining factors associated with disease outcome (e.g. clinical or multi-omics) with RNA data selecting for LINCS/L1000 landmark genes, TargetTranslator constructs patient stratifying *signatures*; 2) These signatures are scored for consistency across model cell-lines and across data cohorts. Significance is assessed via FDR-controlling permutation testing; 3) The scoring results are combined with network data to map risk factors to drug targets. TargetTranslator can accommodate several types of omics data in step 1, such as DNA copy number aberrations, miRNA or DNA methylations, as long as one of the data types is gene expression data, which allows for mapping of signatures onto LINCS/L1000 gene space.

1. Signature construction. Step 1 of TargetTranslator maps multi-omics, or clinical data, relating to disease outcome onto RNA data comprising the L1000 (or any other) landmark genes. In this step, we can combine data from several studies or cohorts for joint analysis. Specifically, we denote the disease data matrices as $\mathbf{Y}(i)$ and the RNA-matrices as $\mathbf{Z}(i)$, for cohorts $i = 1, 2, \ldots$. The factors associated with disease outcome have dimensionality $dim(\mathbf{Y}(i)) = p_{disease} \times n_i$, where the same set of $p_{disease}$ factors are considered to stratify patients in terms of risk across all cohorts. The RNA data is of dimension $dim(\mathbf{Z}(i)) = p_{LINCS} \times n_i$.

We next consider how to extract low-dimensional features from the disease outcome factors. Below we outline the case where the matrix Y comprises 'omics data (e.g. gene expression for a set or risk genes or pathway). At the close of the section we briefly discuss how TargetTranslator can also utilize a supervised feature extraction to handle clinical data like survival. We assume that the outcome factors can be summarized with a low-rank feature such that

$$\mathbf{Y}(i) = \mathbf{H}(i)\mathbf{F}(i) + \epsilon_Y(i), \tag{1}$$

where $dim(\mathbf{H}(i)) = p_{disease} \times k, dim(\mathbf{F}(i)) = k \times n$. $\mathbf{F}(i)$ represents a k-dimensional feature across patients in cohort i that summarizes the patient variability across patients with respect to the $p_{disease}$ outcome associated factors. TargetTranslator extracts these features through a low-rank matrix decomposition (SVD). Next, we project data $\mathbf{Z}(i)$ onto the extracted features $\mathbf{F}(i)$;

$$\mathbf{Z}(i) = \mathbf{B}(i)\mathbf{F}(i) + \epsilon_Z(i) \tag{2}$$

where$dim(\mathbf{B}(i)) = p_{LINCS} \times k, dim(\mathbf{F}(i)) = k \times n_i$. The matrix $\mathbf{B}(i)$ are the $k - dimensional$ *signatures* for the L1000 landmark genes. These are obtained through the regression

$$\widehat{\mathbf{B}}(i) = \mathbf{Z}(i)\mathbf{F}(i)^T(\mathbf{F}(i)\mathbf{F}(i)^T)^{-1}. \tag{3}$$

The relationship between the different matrices in our model is illustrated in (**Supplementary Figure 2**).

The TargetTranslator tool has been developed to handle a rich set of data for stratification of patients, including multi-omics data such as The Cancer Genome Atlas. Due to its adaptable formulation, it can be run using an arbitrary number of 1 or more stratification variables or risk factors, providing a flexible tool to explore the data. This means that the method makes no principal difference between extracting profiles across, for instance, a single mutation, a group of methylation events, or specific clinical variables. Special cases like a single binary variable (e.g. mutation) or a single continuous variable (e.g. pathway score) do not require the first dimension reducing step and the second step thus corresponds to a discriminant score and correlation, correspondingly.

TargetTranslator can also be run in a supervised setting; here, the user provides the latent variables $\mathbf{H}(i)$, typically

as gene signatures, whereby $\mathbf{F}(i)$ is found by least squares. In another special case, the method can accommodate left-censored survival data. For this, the feature F is defined to be the log proportional hazard of each patient, as obtained by a Cox regression model.

2. Signature scoring. Next, we are concerned with the matching between the signatures $\widehat{\mathbf{B}}(i), i = 1, 2, ...$ and the full LINCS compendium. We have organized and normalized the full LINCS data into a 3-dimensional table, in which the p_{LINCS} rows are genes, columns $C = 19763$ are drugs (or shRNAs) and $N = 14$ layers are cell lines. We use $\mathbf{g}_{r,s}$ to denote the (z-transformed) gene expression vector for drug r in cell line s and the function $\sigma(\mathbf{g}, \mathbf{h}) = \sigma(\mathbf{g}^T\mathbf{h})$ to denote the similarity between any two profiles, where $\mathbf{g}^T\mathbf{h}$ is the scalar product between $\mathbf{g}$ and $\mathbf{h}$. The explicit form for $\sigma(\cdot, \cdot)$ is obtained as follows;

We construct a training data set from the LINCS data comprising two classes: (i) pairs of gene expression profiles representing the same drug but in different cell lines (class 1) vs (ii) randomly selected pairs for profiles (class 0) (c.f. **Supplementary Figure 1A**). We then fit a logistic regression to the training data. Thus, the distance metric used here is a logistic function of the scalar product between **g** and **h**:

$$\sigma(g, h) = \frac{1}{1 + e^{-(u+v(\mathbf{g}^T\mathbf{h}))}}, \tag{4}$$

with parameters u, v are obtained from the logistic regression.

As a compound score for a particular perturbation (drug), we compute the average σ value when comparing that drug across N different LINCS/L1000 cell lines:

$$\bar{\sigma}(r) = (\frac{N(N-1)}{2})^{-1} \sum_{s<s'} \sigma(g(r, s), g(r, s')), \tag{5}$$

which can be interpreted as the propensity for drug r's profile to match across different model cell lines. Creating a drug-specific matching score from the LINCS data captures the fact that some drugs separate into match/non-match while others do not or poorly so. We need to take this into account when computing match scores against the TargetTranslator signatures.

Thus, finally, we compute the aggregate score of a perturbation r, defined as:

$$S(r) = \bar{\sigma}(r) \frac{1}{N} \sum_{s=1}^{N} \sigma(\mathbf{g}_{r,s}, \pm\mathbf{B}(1)) \times \sigma(\mathbf{g}_{r,s}, \pm\mathbf{B}(2)) \times \ldots, \tag{6}$$

where $\pm\mathbf{B}$ denotes that the score is computed either with respect to $-1 \times \mathbf{B}$, to detect drugs that suppress the signature, or $+1 \times \mathbf{B}$, to detect drugs that enhance the signature. This score weighs the drug-specific propensity for signatures to match across cell lines with the average (expected) similarities between LINCS profiles and the TargetTranslator signatures $\mathbf{B}(i)$ across *all cohorts*. In the cases were LINCS/L1000 $\mathbf{g}_{r,s}$ is unavailable for a specific value of s, the average above is computed for the cell lines with available data, with N adjusted accordingly.

This scoring function has three key properties. First, since each of the σ terms has a value between 0 (no match) and 1 (match at a level expected for the same target), the aggregate score will be on the interval between 0 and 1. Second, the $\bar{\sigma}$ term specifically serves the purpose of giving a stronger weight to any perturbation that gives a consistent response across model cell lines. Third, since a product is formed between the σ values across all

cohorts, this score will emphasize matches that are consistently observed across cohorts. It is possible to consider variations of this matching function, in particular differential weights to the cell lines, depending on the biological question. This is reserved for future work; as a case example, however, we performed an analysis in which our 384 well gene expression profiling was compared to LINCS/L1000 cell lines, using linear regression to fit a weighted mixture that best approximates the results in our NB-PDX model. The average regression weights were higher for the two neural cell line (NPC, NEU) in the cell line panel used (**Supplementary Figure 4**).

To assess the significance of matching scores, we compute the null distribution of S scores using a permutation strategy. TargetTranslator step 1 is run on data for which the order of the patient cases is permuted when building the signatures **B**(i). The FDR of a particular perturbation r is thusly defined as the fraction of permutation distribution scores with a value higher than $S(r)$ (c.f. Figure 4).

3. Target deconvolution step. For target deconvolution, we consider the relationship between the aggregate scores $S(r), r = 1, 2, \ldots$ of the different perturbations; and, the pathway context or target of each perturbation. For a given protein target, we use the data in STITCH to divide all perturbations into two sets: R+ and R-. The set R+ contains all perturbations with a STITCH score greater than 900 (this is considered a stringent hit, whereas 700 is significant (1)). The set R- contains all perturbations below that threshold. We subsequently apply a two-sample Kolmogorov-Smirnov (KS) test to test the sample $\{S(r), r \in R+\}$ vs the sample $\{S(r), r \in R-\}$. P-values are corrected by mafdr to obtain FDR q-values. Note that this test is invariant to whether drugs are antagonists or agonists. When scoring for negative matches, an antagonist will have a score close to 1, and an agonist will have a score close to 0. Either will contribute to a low p-value of a KS test, since both low (close to 0) and high (near 1) values will shift the cumulative distribution distance that underlies the KS test. Similarly, when scoring for positive matches, agonists will have match scores close to 1 and antagonistic be close to 0.

TargetTranslator package and web tool. TargetTranslator is accessed via targettranslator.com and the R package targettranslator is available upon request.

Preparation of data and signatures for neuroblastoma target identification

1. Preparation of Neuroblastoma data sets and signatures.

NB tumour gene expression data from collections, which we denote R2, TARGET and SEQC:

- R2: Transcriptomes of 88 neuroblastoma measured on the Affymetrix Human Genome U133 Plus 2.0 Array (2), GEO GSE16476 (log2 gene-level expression values)
- TARGET: transcriptomes of 247 NB tumours measured on the Affymetrix Exon ST array. from the NIH TARGET program, ftp://caftpd.nci.nih.gov/pub/OCG-DCC/TARGET/NBL/gene_expression_array/L3/ (log2 gene-level signals)
- SEQC: transcriptomes of 498 NB tumours measured by RNA sequencing (3), GEO GSE49711, representing log(FPKM+1) transformed gene expression signals.

From the annotation of these data, we obtained information about sex (M/F), age, INSS and COG group, survival data (observation time and vital status), *MYCN* amplification (amp/not amplified); and (in R2 and TARGET) 11q deletion, 17q gain, *ALK* mutation. Gene signature definitions were obtained from published sources and the Molecular Signatures Database (MSigDB), with marker genes as listed in (**Supplementary Data 1**).

In the first step of our analysis (Signature construction, equations 1-3 above), these data were used to estimate neuroblastoma disease signatures in L1000 gene space. Depending on the type of disease information, this was done as follows:

- **Univariate disease information (e.g. *MYCN* amplification)**. Consider first the specific case of *MYCN* amplification. In this case, the disease factor of interest (denoted as the F matrix above) is simply *MYCN* status in each of the patients, mathematically represented as a 1xn matrix, where n is the number of patients, containing *MYCN* status is encoded as 0=non-amplified, 1=amplified. The above RNA data sets are arranged into 3 different matrices, Z(1), Z(2) and Z(3), with L1000 genes as rows (log expression, row-centered), and columns corresponding to the same (matched) patients as in the F matrix. Application of equation 2 and 3 then gives the signature for each of the RNA data sets, denoted as B(1),B(2), and B(3). This basic procedure was used to estimate B signatures for sex, age, COG, INSS, *MYCN*, 11q deletion, 17q gain and *ALK* mutation(i.e. all the cases where the disease information is univariate).
- **Multivariate disease information (e.g. differentiation gene signatures)**. In a second case, the disease information is multivariate and represented by the matrix Y, in which rows are the signature genes, columns are patients, and elements contain log RNA expression levels of the signature genes in the patients (row-centered). In this case, we use equation 1 to estimate F as the rank 1 approximation of Y, followed by estimation of B(1), B(2) and B(3). (Higher ranks than 1 are possible, and will be explored in future work). This extended procedure was used to estimate the B signatures for each of the signatures (**Supplementary Data 1**).
- **Survival information.** In the third case, the disease information consists of survival data. Here, we applied a standard Cox proportional hazards model, with 7 relevant covariates (Y1=age, Y2=COG, Y3=INSS, Y4=11q deletion, Y5=17q gain, Y6=*MYCN* and Y7=*ALK* mutation). The Cox model is expressed as h(t) = h0(t)exp(β1Y1 + β2Y2 + …), where h(t) is the hazard function, h0(t) is the baseline hazard, Y represents the covariates, and β terms represent coefficients. After fitting this model (Matlab coxphfit), the log risk score F for each patient is given by F=β1Y1 + β2Y2 + … + β7Y7. Given F, application of equation 2 and 3 then gives the signature for each of the RNA data sets.

2. Preparation of LINCS/L1000 data.

LINCS/L1000 was downloaded from GEO (GSE92742) in its level 3 version containing quantile normalised expression values (file GSE92742_Broad_LINCS_Level3_INF_mlr12k_n1319138x12328.gctx, GSE92742_Broad_LINCS_inst_info.txt, GSE92742_Broad_LINCS_gene_info.txt) As described in our previous work, we normalized the L1000 data using the RUV (Remove Unwanted Variation) framework, which - when applied in this context - aims to remove plate effects and bias not removed by basic strategies (4; 5). In its general formulation, the RUV models gene expression data Y as:

$$\mathbf{Y} = \mathbf{X}\beta + \mathbf{W}\alpha + \epsilon, \tag{7}$$

where **Y** is the samples by genes matrix of log L1000 expression data. **X**, in turn, is an observed matrix whose columns are the factors of interest (e.g. drug, cell line, time point, dose). β are the coefficients to be estimated and will contain the effect of each factor (e.g. drug) affects gene expression. W is a matrix of k unobserved covariates and is used to represent the unwanted variation (e.g. plate, batch). W has dimension samples by k, where k is set by the user (see below). To fit the model, we used the RUV-2 algorithm, which operates in two steps. First, the user defines a portion of the data which contains the unwanted variation Y_0. Improving on our previous pipeline, which depended on housekeeping genes, we here used vehicle (DMSO) controls of L1000, which are abundant in the data set, and present on each plate and experimental batch. Given the Y_0 matrix, RUV-2 uses factor analysis to estimate the unwanted bias term W α. With this term known, β is fitted using standard least squares. We processed data for each of the LINCS/L1000 cell lines separately. For each cell line, we produced 5 versions of

the data.

- Pooled data. All drug-treated instances and DMSO control for that cell line.
- 6 hours treatment, dose $\geq 10\mu M$
- 6 hours treatment, dose $< 10\mu M$
- 24 hours treatment, dose $\geq 10\mu M$
- 24 hours treatment, dose $< 10\mu M$

For each of these cases and each L1000 cell line, we used the RUV-2 procedure to estimate the matrix of drug effects. Since the effects are estimated from log-transformed RNA expression data (L1000 level 3) and normalized against DMSO controls, they represent drug-specific log fold expression profiles of each drug relative to the vehicle. The number of drugs used in each L1000 cell line differs, and that out of 77 cell lines, we retained the 14 cell lines for which the data contain results for at least 1000 unique compounds (A375, A549, ASC, HA1E, HCC515, HEPG2, HT29, MCF7, NEU, NPC, PC3, PHH, SKB, VCAP). As explained below, our benchmarking results clearly indicated that the pooled version of the data performs better, which is consistent with (4). The pooled version was therefore used in Figures 1-3. The choice of k (RUV rank), which is an open parameter in the pipeline, was guided by inspection of data matrices (c.f. (4)) and our benchmarking cross-validation, in which we measured the ability of TargetTranslator to detect drugs with the same target, discussed below and in (**Supplementary Figure 1**).

3. Preparation of STITCH data.

We downloaded the STITCH database as a flat file from stitch.embl.de (9606.protein_chemical.links.v5.0.tsv.txt). STITCH was then linked to L1000 in two steps. First, chemical identifiers were subsequently matched using their International Chemical Identifier (InChI) keys, (available in the STITCH data file chemicals.inchikeys.v5.0.tsv.txt and the Broad data file GSE92742_Broad_LINCS_inst_info.txt, respectively). Second, STITCH protein identifiers (ENSP numbers) were mapped to corresponding gene symbols (HGNC) using tables downloaded from Ensembl BioMart (https://www.ensembl.org/biomart). The results were re-arranged as a matrix $H = \{H_{ij}\}$ with rows corresponding to targets (denoted by their gene symbols), columns corresponding to drugs in L1000, and each element H_{ij} representing a STITCH association between that target and that L1000 drug. In cases where the STITCH score was 900 or higher (strong association according to STITCH),$H_{ij} = 1$, otherwise $H_{ij} = 0$. The STITCH data was further checked by benchmarking simulations, discussed below.

4. Other data. RNAi DEMETER2 scores for **Supplementary Figure 4G** were obtained from the Broad Institute dependency map project (depmap.org) downloads as D2_combined_gene_dep_scores.csv. Further information on DEMETER2 is available on https://depmap.org/R2-D2/.

Method and data benchmarking.

1. Benchmarking of signature consistency across neuroblastoma data sets.

As a first evaluation, we analysed the consistency of gene signatures from three different neuroblastoma cohorts, termed R2 (88 cases), TARGET (247 cases) and SEQC (498 cases). The analysis was primarily aimed at determining which aspects of neuroblastoma biology (e.g. *MYCN* amplification) could be represented as consistent RNA profiles with the available data, across all cohorts. As a secondary objective, we investigated the effect of replacing the above estimation method by a different method (ChDir). To answer these questions, we computed three RNA signatures B1, B2 and B3 (one for each cohort). As the evaluation metric, we used the average Pearson correlation between all pairs of B1, B2 and B3. If all three cohorts are fully consistent, this value should be close to 1 and if the profiles are uncorrelated the value should be close to 0. Profiles were consistent between cohorts

for most of the signatures evaluated, such as *MYCN*, showing that the data were of good quality (**Figure 1**). We also noted that some aspects were less well represented as RNA signatures, which can reflect factors such as the number of cases carrying a mutation (e.g. *ALK*) or a more complex relationship between the process and RNA levels in the tumour (like age).

2. Benchmarking of pooled L1000 data vs single doses and time points.

As a second evaluation, we analyzed if our assembled L1000 and STITCH data supported the identification of drug targets from signatures. The benchmarking aimed to address three questions. Firstly, we sought to measure the effect of pooling data (using the RUV model) across time points and doses. Second, we sought to relate the TargetTranslator score to a state-of-the-art metric, RGES (6). Last, we sought to define a suitable value for the RUV rank parameter. We defined a benchmarking set of drug targets for which the L1000 contained at least 5 compounds (n=57). For this set of targets, we executed simulation:

1. For each target, identify the set $D = \{d_1, d_2, ...\}$ of L1000 drugs with a STITCH link to that target. For each drug d_i in D, compute its match score with all other compounds in the L1000 data matrix. Given all the match scores, record the relative rank of all other drugs ($d_j \in D$, $i \neq j$), where relative rank = 0% for highest rank and 100% for the lowest rank.
2. Assemble all relative ranks into a histogram, showing the distribution of ranks for drugs with that target.
3. Summarize the performance as the average histogram, shown in Supplementary Figure 1A-B.
4. Repeat for different versions of the L1000 data matrix (pooled, 6/24 hours, high/low dose, see above), different match scores, and different values of the RUV parameter.

Pooled times and doses using the RUV model gave higher enrichment of low ranks than individual dose/time subselections of L1000 (c.f. (6)) (**Supplementary Figure 1A**). TargetTranslator gave higher average (across targets) enrichment at low ranks than RGES (**Supplementary Figure 1B-C**). The choice of the RUV parameter k had a modest effect on performance but was stable over a range of values (**Supplementary Figure 1D**).

3. Benchmarking robustness of STITCH data.

As a third evaluation, we investigated whether TargetTranslator results are affected by the completeness of the drug target matrix (STITCH data, summarised as the H matrix, described above). The homo sapiens part of STITCH contains 15,473,940 associations between proteins and chemical compounds (1). Mapped to the LINCS/L1000 data, the STITCH data contains 452,782 links between human proteins and chemical compounds. It is highly likely that these data are incomplete, in the sense that some drug-to-target associations are missing. To understand the impact of missing associations, we repeated the analysis in the main manuscript TargetTranslator for both (i) the full STITCH data, and (ii) subsampled versions of the STITCH data, in which a specific random percentage of links had been removed (set to 0 in the H matrix). With the full STITCH data, 87 targets were predicted (Figure 3). After 10% removal, 86 targets were retained (loss of JUN). After 25% STITCH data removal, 82 targets were found (loss of AURKA, CHRM4, FLT3, JUN, PGR). When removing an extreme 50% of STITCH data, 57 targets were retained (30 targets lost). Thus, our test is robust to missing data in STITCH, up to 25% deletion. The two reported targets, MAPK8 and CNR2 remained even after deleting 25% (MAPK8) and 50% (CNR2) of links. We expect our method, and other frameworks based on STITCH, to give stronger results as more data become available in future STITCH releases.

Compounds. High scoring drugs chosen for validation were PI3K inhibitor omipalisib, mTOR inhibitor torin-2, CDK4/6 inhibitor palbociclib, CDK1/2/9 and GSK3β inhibitor AZD-5438, PPARγ agonist rosiglitazone, Rho-kinase inhibitor fasudil (Selleckchem), ceramide analog DL-PDMP (Santa Cruz Biotechnology), cannabinoid receptor

2 (CNR2) agonist GW-405833 hydrochloride, peripheral benzodiazepine receptor (PBR) antagonist PK-11195 (Sigma-Aldrich), MAPK8 inhibitor AS-601245 (Abcam), and HMG-CoA inhibitor lovastatin (Enzo). JQ1 (Selleckchem) and retinoic acid (Sigma-Aldrich) were used as positive controls for N-Myc downregulation and differentiation respectively. SR144528 (Sigma), GW842166X, Otenabant (SelleckChem), ACEA, HU308, JWH133 (Tocris) were used to study CNR1/2 engagement. CC-930, SP600125, D-JNKI-1, JNK-IN-8 (MedChemExpress) were used to study MAPK8,9,10 engagement. All compounds were dissolved in DMSO, Ethanol, or MilliQ water according to the vendor's instruction to a stock concentration of 1-20 mM. Mouse studies were conducted using GW405833 (Cayman Chemicals) and AS601245 (MedChemExpress), dissolved in DMSO to 60 mg per ml and 120 mg per ml, respectively, and diluted in a vehicle containing 11 % ethanol and 0.25 % Tween20 in a 0.9 % saline solution, with a maximum end concentration of 5 % DMSO upon injection.

Cell culture. Two high-risk patient-derived xenograft (PDX) cell lines, NB-PDX2 and NB-PDX3, developed at Lund University Hospital (termed LU-NB-2 and LU-NB-3 in previous publications), were cultured on laminin (LN) 521 (Biolamina) coated primaria plates (VWR) in defined neuroblastoma media containing 40 ng per ml bFGF and 20 ng per ml EGF (7; 8). NB-PDX2 derives from a cerebral metastasis of a stage 4 neuroblastoma patient and NB-PDX3 derives from a primary tumour in the adrenal gland of a stage 3 patient. The molecular data for both cell lines include *MYCN* amplification, 1p loss and 17q gain. Neuroblastoma cell lines SK-N-BE(2) and SK-N-SH were cultured using the same defined neuroblastoma media on Primaria plates without laminin-coating. Glioblastoma cell line U3013MG was kindly provided by the Human Glioblastoma Cell Culture (HGCC) consortium (hgcc.se) and was used to distinguish between neuroblastoma specific and general anti-tumour activity. U3013MG was cultured under stem cell conditions in neural stem cell (NSC) medium containing bFGF and EGF (9; 10). All cells were cultured under 5 % CO2 pressure at 37 degrees C to maximum confluency of 80 % and detached using StemPro Accutase (ThermoFisher Scientific) when passaging. Cells were tested for mycoplasma contamination on a regular basis.

Viability. 5,000-10,000 cells were seeded on the day before treatment as described above and treated for 72 h using a range of drug concentrations including the TargetTranslator predicted concentration. Viability was detected using the metabolic activity assay AlamarBlue (Invitrogen) and performed according to the manufacturer's protocol. Results were calculated as a viability ratio to vehicle control. A Sigmoidal 4PL curve with a bottom constraint of y=0 was fitted using GraphPad Prism version 6.05 for visualization and to extract doses corresponding to the 10%, 20%, 50% and 80% effective concentrations (IC10, IC20, IC50, and IC80 respectively) for following experiments. When an IC50 value could be obtained from the software for most of the cell lines (GW405833, AS601245, fasudil, AZD5438, rosiglitazone, PK11195, and lovastatin), differential IC50 concentration between neuroblastoma and glioblastoma cells was calculated instead. This was done by a one-way ANOVA and Dunnett's multiple comparisons test by comparing all neuroblastoma cell lines against U3013MG (GraphPad Prism version 6.05) at a specific dose, which is indicated by an arrow in Figure 5A. For target-engagement follow up, 10,000 NB-PDX3 cells were seeded on the day before treatment, treated with compounds targeting MAPK8/9/10, CNR1, or CNR2 for 48 h and viability assessed using AlamarBlue as described above. For GW405833 rescue experiments, cells were seeded in a similar way on the day before treatment, pretreated for 30 min with CNR1 or 2 antagonists or agonists before adding GW405833 in a combination treatment. Viability was assessed using AlamarBlue.

Pharmaco-transcriptomics. Cells were seeded one day prior to treatment using a Multidrop 384 liquid dispenser (Thermo Scientific) in LN-521 coated 384-well microplates (BD Falcon Optilux #353962), at a density of 2000 cells per well. Each of the 11 compounds, retinoic acid, JQ1, or DMSO were transferred from a PP384 plate (Labcyte) using an Echo"R 550 non-contact liquid handler (Labcyte) to a V-bottom polypropylene plate (Greiner # 781280) for dilution in NSC medium before dispensing into cell plates using the MDT 384 head on a Janus automated

workstation (PerkinElmer). Following either 6 (plate 1) or 24 hours (plate 2) treatment, the drug-containing medium was removed and the plates were snap-frozen until further processing. The final plate layout consisted of 16 treatments (13 compounds, 2 DMSO concentrations, 1 untreated cell medium) x 3 doses (equivalent to IC50, IC20 and IC10) x 4 replicates x 2 cell lines (total 384).

The RNA-Seq libraries were prepared in a 384-well plate format using the SMART-Seq2 method, (11). After the completion of the library preparation, equimolar amounts of 384 libraries were multiplexed together in one pool and then sequenced by an Illumina HiSeq 3000 for 51 sequencing cycles, targeting around 0.8 million reads/well as an output. The raw single-end sequences were quality-controlled using FastQC v.0.11.4 (http://www.bioinformatics.babraham.ac.uk/projects/fastqc/), the low-quality bases (quality scores < 30) and the adaptor contamination (if present) were removed by Trimmomatic v.0.36 (12). In the quality control step, we eliminated biological replicates (wells) with low sequencing output. The high-quality reads were mapped by STAR v.2.0.5 (13) against the Ensembl reference genomes Homo sapiens (GRCh38, ftp://ftp.ensembl.org/pub/release-90/fasta/homo_sapiens/dna/). The uniquely mapped reads aligned to exons were counted by HTSeq v.0.6.1 (14), then normalized by the DESeq2 R package v.1.14.1 (15).

PCA, ROC curves and GSEA. The PCA plot in Figure 3 was computed from drug-induced log fold change values, pooling replicates for each dose. We also used linear regression to model the position along each principal component axis (c.f. the X and Y axes in Figure 3) as a function of dose and time point as covariates. **Supplementary Table 1** shows the significance of dose and time effects for each drug and each component. Note that negative controls show no association while all drugs show association for at least one effect, if not several (**Supplementary Table 1**). ROC curves in Figure 4 were based on comparison of (i) the correlation between neuroblastoma risk signatures and each drug's average fold change signature in L1000 (here seen as the prediction) and (ii) the correlation between neuroblastoma risk signatures and each drug's corresponding average fold change signature in our RNA-Seq experiment in PDX cells (here seen as the ground truth). ROC curves (x=1-specificity of prediction in relation to ground truth; y=sensitivity) and AUC integrals of ROC curves were computed using Matlab's roc.m and trapz.m functions. Gene Set Enrichment Analysis (GSEA) was performed using the GSEAPreranked option in the GSEA software (version 2.0.9) (16). For each compound, a ranked list was prepared by running differential expression analysis on transcriptomic data from treated and control samples using the DESeq2 R package v.1.14.1 (15). GeneIDs and log2 fold change values were extracted from the analysis output and used to create the ranked list. These were then submitted to GSEA in pre-ranked mode using default parameters.

Western Blot. 400,000 - 500,000 cells were seeded in LN-521 (Biolamina) coated 6-well Primaria plates (VWR), treated for 48 h with approximated EC50 concentrations of positive control JQ1 1-6 μM, positive control retinoic acid 80-120 μM, and explored compounds GW405833 1.4-6 μM, fasudil 35-50 μM, AS601245 4.5-7 μM, lovastatin 2-4 μM, rosiglitazone 39-93 μM, DL-PDMP 24-50 μM, AZD5438 0.53-0.6 μM, omipalisib 3-20 nM, palbociclib 66-100 n, or torin 2 0.035 μM. After 48 h, cells were lysed with RIPA buffer (ThermoScientific) and 25 μg of protein was separated on a NuPAGE 4-12% bis-tris gel (Invitrogen) and transferred using the iblot Gel transfer Stacks nitrocellulose mini kit (Invitrogen) on an iblot®gel transfer device (Invitrogen). Membranes were blocked with StartingBlocking T20 TBS (ThermoScientific) and stained with antibodies against the N-Myc (#ab16898, Abcam, 1:250) and with cyclophilin B (#ab16045, Abcam, 1:500) as a loading control. N-Myc protein expression (n=6) was normalized against cyclophilin loading control. Significance level was calculated using a one-sample t-test with Benjamini-Hochberg false discovery rate (FDR) correction.

Apoptosis. 10,000 NB-PDX2 or -3 cells were seeded on LN-521 coated 96-well Primaria plates and treated with 0.1 % IncuCyte® Caspase-3/7 Green Reagent for Apoptosis (#4440) according to manufacturer's protocol. Cells were treated with palbociclib 0.1 μM, lovastatin 3 μM, fasudil 50 μM, GW405833 6 μM, omipalisib 0.02 μM,

AZD5438 7 μM, JQ1 3 μM, DL-PDMP 50 μM, retinoic acid 120 μM, PK11195 90 μM, torin 2 0.04 μM, DMSO 0.5 %. Phase-contrast and fluorescent images were acquired over 96 h with the Incucyte S3 instrumentation (Essenbio). Time-lapse confluence and apoptosis were automatically masked using the built-in software and apoptotic response was quantified as apoptotic area (μm^2 of image) per confluence (% of the image) and shown as a ratio to vehicle control. Statistical significance was assessed using one-way ANOVA with Dunnett's multiple comparison test (GraphPad Prism version 6.05).

Image analysis of confluence and neurite outgrowths using adherent cell imaging. The effect of different doses (seven-point dose scale) of each of the 11 compounds, retinoic acid, or DMSO 0.01% on confluence and neurite outgrowth was followed in real-time using an IncuCyte Zoom imaging instrument (Essen Bioscience). Phase-contrast images of NB-PDX2 or NB-PDX3 cells (2000 cells/well) plated in LN-521-coated 384-well microplates (BD Falcon Optilux #353962) were taken every 2 h for a total of 3 days. There were two replicate wells for each cell line, dose and compound.

The U-net (17) convolutional neural network (CNN) were used for pixelwise classification of the images, using Keras v.2.2.4 (18), Tensorflow v.1.9.0 (19) and Python v3.4. 64 filters for the top layer and six downsampling layers were used. The CNN was trained on 35 manually segmented images of at least 512 x 512 pixels for 2000 epochs. For each epoch the training data was augmented by: random cropping (in case of images $>$ 512 x 512 pixels), rotation, translation (20 %), shearing (20 %), zooming (20 %) and flipping. Images were normalized by adaptive histogram equalization prior to training and classification. Each pixel was classified as one out of the four classes: background, alive cell, dead cell or cell protrusion. The pixelwise cross-entropy loss was weighted in order to ensure equal class balance. There was no attempt at separating individual cells. The raw images were split into smaller 512 x 512 pixel large images with 48 pixels of overlap. After classification, each split image was assembled by taking the weighted (euclidean distance to image edge) mean of overlapping pixels. The confluence for each image was defined as the number of pixels classified as alive cell divided by total pixels in the image.

We skeletonized the cell and protrusion layers of each image to get a binary representation of the skeleton of the cell-blobs and protrusions. By following the adjacent protrusion pixels of the skeleton we measure the length of each protrusion. Since protrusions may split or cross we measure the longest path for each connected protrusion. Protrusions that did not connect (at either end) to a cell-blob were removed. The number of protrusions of $length > d$ in an image is roughly distributed as an exponential distribution. We fit the function $A \cdot e^{-k \cdot d}$, where A is the number of protrusions, d is the length and k is the decay, to each image. We define:

$$Y_{np} = \frac{A}{N_{bp}} \tag{8}$$

$$Y_{md} = -1000 \cdot k \tag{9}$$

where N_{bp} is the total length of the cell-background interface, Y_{np} is the normalized amount of protrusions and Y_{md} is the *morphological differentiation score* and describes the relationship between long and short protrusions in the image. Cells with long protrusions will hence have a larger *morphological differentiation score*. Refer to **Supplementary Figure 9A-D** for an example of this process. For completeness, we provide a figure for the change in a number of protrusions (**Supplementary Figure 9F**).

Linear mixed effects models were used for the estimation of compound effects and computation of p-values using the lmerTest v.3.0(20) package in R v3.5.1. The lmerTest package uses Satterthwaite approximations of the degrees of freedom to get a more correct and conservative estimate of the computed p-values. For the effect on

confluence, using R formula syntax, we used the model:

$$\text{LogConfluence} \sim 1 + \text{Time} : \text{Celline} : (\text{HasDMSO} + \text{Compound} : \text{Dose}) + (1|\text{Well}) \tag{10}$$

where $Celline$ and $Compound$ are both categorical variables of 2 and 12 items respectively. $HasDMSO$ corrects for the effect of DMSO and is set to one for wells including DMSO and zero otherwise. $Dose$ is categorical and measures the effect of each dose and compound separately. Since a low number of cells lowers the quality of the protrusion estimates we estimated the effect on the protrusions using the dose closest to IC10. The model used is analogous to the confluence model, but where the dose variable is binary rather than categorical. We correct for multiple tests using the Benjamini-Hochberg FDR correction. We correct the confluence p-values using all estimates from the confluence model (n=173). We correct the protrusion estimates using all estimates from both protrusion models (n=58). Residual bootstrapping (1000 bootstraps) was used to create a bootstrapped distribution of the protrusion estimates.

Toxicity in zebrafish embryo after drug treatment. All zebrafish experiments were approved by the regional ethical board (Uppsala Djurförsöksetiska nämnd, C68/15, 5.8.1-08213/2017, and EP 161/14). For the drug toxicity study, 2 days post fertilization (dpf) zebrafish embryos were exposed to drug-containing embryo water containing methylene blue for 24 h and signs of toxicity was observed by the researcher. Drug doses correspond to approximations of the IC20, IC50, and IC80 concentrations based on viability data (see above). The level of toxicity was noted on a numerical scale, where 0 = no toxicity, 1 = noted toxicity in less than 2 fish, but no toxicity noted in the others, 2 = vague toxicity in the majority of fish, no deaths, 3 = high toxicity in the majority of fish, no deaths, 4-5 = lethal doses of drugs, with 5 suggesting that the effect might have been instant.

Treatment of neuroblastoma zebrafish xenografts. NB-PDX3 was transfected with a lentiviral construct containing GFP, luciferase and puromycin resistance genes (pBMN(CMV-copGFP-Luc2-Puro), Addgene plasmid # 80389, a kind gift from Prof. Magnus Essand, Uppsala University). Cells with positive integration were selected under 0.5 μg per ml puromycin treatment for two weeks and high GFP expressing cells were sorted using FACS and cultured as described above. Prior to embryo injection, cells were detached with StemPro Accutase (ThermoFisher Scientific), passed over a 40 μm sterile filter, pelleted and resuspended in neuroblastoma media containing 2 % polyvinylpyrrolidone (Sigma #PVP360) to capture single cells and reduce needle clogging. Cells were kept on ice while 1 day post fertilization (dpf) *casper* zebrafish (21) were dechorionated during 10 min in 1 mg per ml pronase. Glass capillaries (Narishige GD-1) were pulled apart using a Narishige PC-10 to form microinjection needles. Right before use, cells were spun down for 5 min at 2,500 RCF and excess media was removed to acquire highly concentrated cells, which were loaded back into the microinjection needle. Zebrafish were anaesthetized with 0.6 mM tricane. Using a microinjector (Narishige IM-31 or Pneumatic Picopump PV820), 100-200 cells were injected into the midbrain in a controlled volume (<10 nl). Cell number was counted manually at least three times before starting the injections and verified every 50-100 embryo injection to ensure consistency throughout the experiment. (This was done by ejecting a fixed volume onto the floor of the well, followed by manual counting to confirm 100-200 cells). Xenografted zebrafish were kept in E3 media with 25 mM HEPES (E3/H) at 33 $^\circ C$. At 2 dpf, zebrafish were screened for tumour engraftment, manually sorted in 200 μl of E3 water into 96 well plates (1 per well), anesthetized, and imaged dorsally and laterally (10x objective) for GFP baseline signal with a Vertebrate Automated Screening Technology (VAST) BioImager (Union Biometrica) using a LEICA DM6 B microscope and a Leica DFC9000 camera. Treatment was done by diluting drugs in E3/H and replacing all embryo water. At 5 dpf, zebrafish were sacrificed with 2.4 mM tricane and an endpoint image was acquired. As the number of cells per injection might vary (100-200 cells), we controlled for the difference in a number of injected cells by normalizing endpoint GFP signal to initial GFP signal from the tumour cells. Tumour growth was assessed individually for each

zebrafish larva and was defined as fold change of GFP-positive area from day 2 to day 5. For each fish, the tumor involved GFP positive area was automatically quantified by first computing the maximum projection image from the available VAST imager Z-stack, followed by background subtraction and thresholding using Otsu's method (MATLAB graythresh). The GFP positive area was computed for lateral and dorsal projections, for both 2 and 5 dpf. Images in Figure 6 are overlays of the brightfield channel max projection and the corresponding max projection in the GFP channel. The treatment effect was analysed in GraphPad Prism (version 6.05) on tumour growth (mean of dorsal and lateral assessments) with one-way ANOVA followed by Dunnett's multiple comparison test.

Staining of zebrafish sections. Zebrafish embryos were injected as described above, sacrificed at 5 dpf, and fixed in 4 % PFA in PBS for 1.5 h at room temperature, washed in PBS and mounted in hitstogel (ThermoFisher Scientific). Gels were dehydrated in ethanol and xylene, embedded in paraffin and sectioned in 6 um transversal slides. Slides were deparaffinized in xylene and ethanol using standard procedures. Every 5 sections were stained with hematoxylin and eosin using standard procedures. The following slide was used for immunofluorescence co-staining with primary antibodies targeting mouse anti Ki-67 (DAKO #M7240, 1:1000) and rabbit anti NuMa (Abcam, #ab97585) overnight at 4 $^{\circ}C$, and secondary antibodies Alexa fluor 555 goat anti-mouse (Invitrogen #A21424) and Alexa fluor 488 goat anti-rabbit (Invitrogen #A11008) and counter-stained with DAPI (ThermoFisher Scientific, #00-4959-52). Slides were imaged using AxioImager (Zeiss).

Treatment of neuroblastoma mouse xenografts. All mouse experiments were approved by the regional ethics committee for animal research (N 231/14), appointed and under the control of the Swedish Board of Agriculture and the Swedish Court. The mice experiments presented herein were in accordance with national regulations (SFS 1988:534, SFS 1988:539 and SFS 1988:541). For the xenograft study, SK-N-BE(2) cells (15×10^6) were injected subcutaneously into the flanks of immunodeficient nude mice (female 5-6 weeks old NMRI-nu/nu, Taconic). At palpable tumour, with a volume of 0.20 cm^3 or greater, the mice were randomized to receive either GW405833 (45 mg per kg, n=10), AS601245 (90 mg per kg, n=8) or vehicle (n=12) intraperitoneally for eight days, or for two-eight days. Due to unexpected toxicity, 5 mice in the AS601245 treatment arm were terminated before day 8 (**Supplementary Figure 6**). Tumors were measured daily, and tumor volume was calculated as $(\text{width})^2$ x length x 0.44. Animals were maintained at a maximum of six per cage and given sterile water and food ad libitum. The animals were monitored for signs of toxicity including weight loss. At sacrifice tumours were dissected in smaller parts and either frozen or fixed in 4 % PFA, for further analyses.

The estimation of drug effects on tumour growth was computed using a linear mixed effects model (R v3.5.1 with package lmerTest v.3.0(20)). The lmerTest package uses Satterthwaite approximations of the degrees of freedom to get a more correct and conservative estimate of the computed p-values. The presented p-values are corrected for multiple comparisons by using Bonferroni correction (n=2). We used the model, in R formula syntax:

$$V \sim 1 + t + t : \text{GW405833} + t : \text{AS601245} + (1 + t + t : \text{GW405833} + t : \text{AS601245} | \text{Mouse}) \tag{11}$$

where V is log tumor volume, t is time; GW405833 and AS601245 are binary variables set to 1 when respective treatment was used.

Histological analysis and quantification of mouse xenograft tumors. Paraffin-embedded mouse samples from all treatment groups (n=3-5) were cut into 6 μm sections and stained with hematoxylin and eosin (HE) or immunostained against proliferation marker MKI67 (#M7240, DAKO) and apoptosis marker cleaved PARP (#5625, Cell Signal Technology) using a standard immunohistochemistry protocol. Antigens were retrieved in citric acid-based buffer (#H-3300, Vector Laboratories) containing 0.05% Tween-20 in 2100 Antigen Retriever (Aptum Biologics Ltd.,), sections were blocked with Animal-free blocking solution (#15019, Cell Signal Technology), primary

antibodies were diluted in Normal antibody diluent (Immunologic), BrightVision Goat Anti-Rabbit or Anti-Mouse HRP Polymer (Immunologic) was applied and signal visualized using BrightDAB (Immunologic). Sections were counterstained with Mayer's hematoxylin (Histolab) and mounted with Pertex (Histolab). Slides were scanned using Nanozoomer S60 (Hamamatsus) and representative images were taken with 40x magnification. The number of positive nuclei was assessed using immunoRatio (ref VJ Tuominen - 2010) plugin for ImageJ (NIH). From each section 8-15 images were taken, covering at least 50 % of the total section area. Results were presented as a percent of positive nuclei.

Data Availability

The sequencing data that support the findings of this study have been deposited in the Gene Expression Omnibus (GEO) with the accession code GSE120920 [https://www.ncbi.nlm.nih.gov/geo/query/acc.cgi?acc=GSE120920]. The source data underlying Figure 4G, 5, 6B, 6E, 7 and Supplementary Figures 4, 5, 6, 9, are provided in the Source Data file. Signatures used in the manuscript are defined in Supplementary Data 1. Additional data files, including the processed LINCS/L1000 and STITCH data can be accessed at targettranslator.org/downloads. For information on materials, contact SN (sven.nelander-at-igp.uu.se).

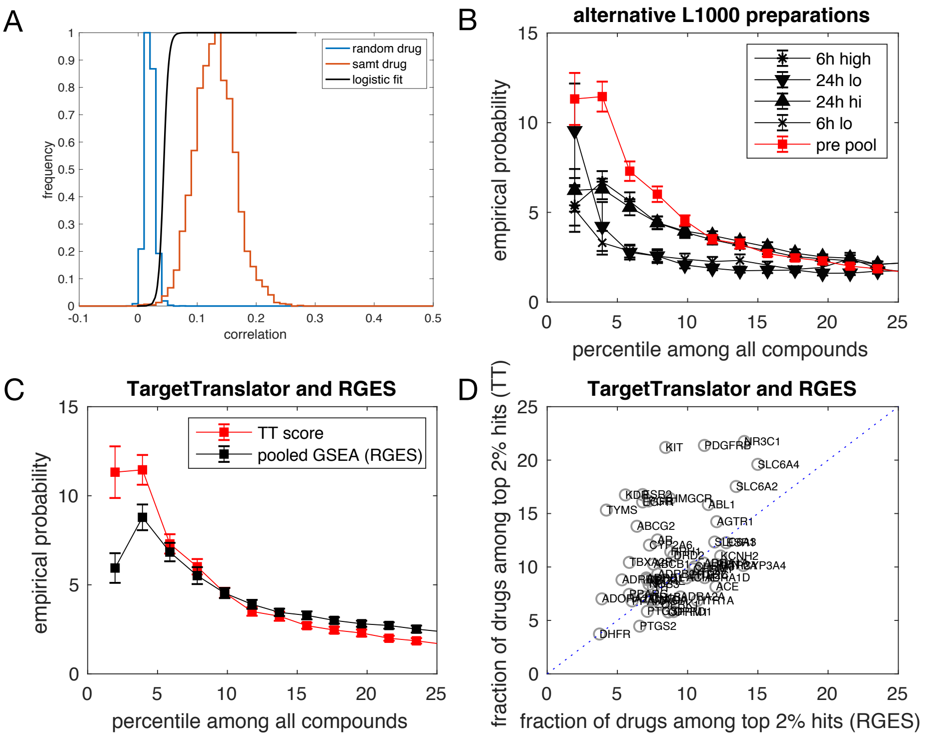

Supplementary Figure 1: Benchmarking of TargetTranslator (A) Logistic regression of FC1000 correlations. Red curve = histogram of correlations between cell lines for the same perturbation. Blue curve = corresponding histogram for randomly chosen perturbations. Dashed line: logistic fit, separating classes of random perturbation vs the same perturbation. (B-D) Benchmarking of TargetTranslator. In a cross-validation (details in Supplementary Methods), we selected drugs in L1000 with a known protein target. Using such drugs as the query, we applied the TargetTranslator match score to rank all other drugs based on RNA profile similarity. We then computed the probability (Y) to find other drugs with the same target at low-ranking (left on the X axis) positions. This was repeated for all 51 targets with at least 5 specific (STITCH score greater than 990) drugs in L1000, to yield an average curve (error bars are 95% CI). (B) We first compared how the choice of subsets from the L1000 data (different doses and time points) affected the performance curve. The best results were obtained when pooling data for the same drug across doses and time points. (C) Next, we compared TargetTranslator with pooled data, to a state-of-the-art matching function, RGES (6) that uses pooled data. Note the higher enrichment by TargetTranslator at low ranks. (D) Last, we compared the probability of compounds with the same target to be among the top 2% for each of TargetTranslator and RGES. The results were comparable, but a subset of targets are better captured by TargetTranslator.

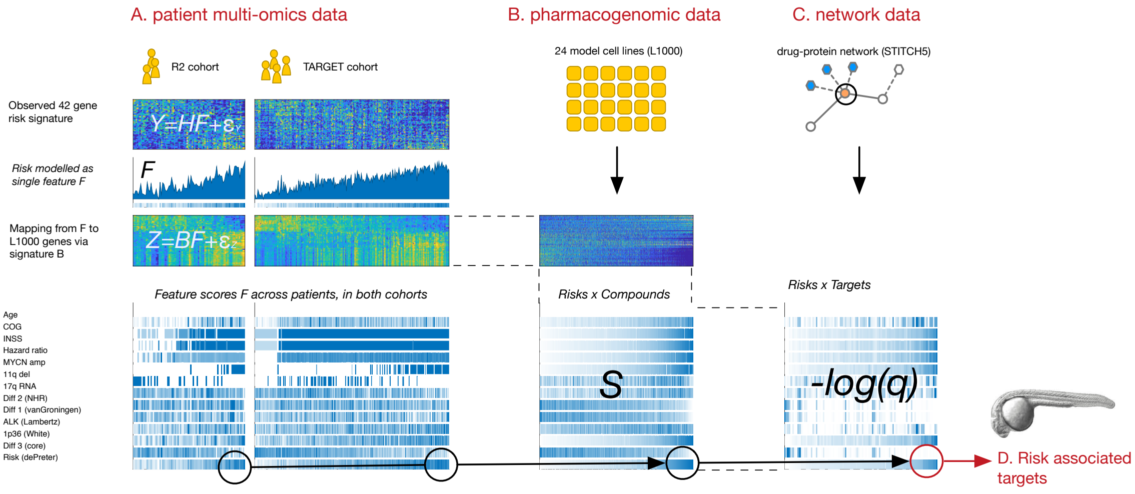

Supplementary Figure 2: Overview of matrices used by TargetTranslator. Y is the part of the patient data that is used to assign risk. The relationship between risk and the expression of L1000 landmark genes in each patient, is modeled by a set of linear matrix equations, where B is the gene signature. By comparing B to L1000 data, we obtain scores (S) for all disease outcome factors / signatures and drugs. The scores are tested against target databases to yield association q-values (heatmap shows -log(q)). Dashed lines indicate shared matrix dimensions.

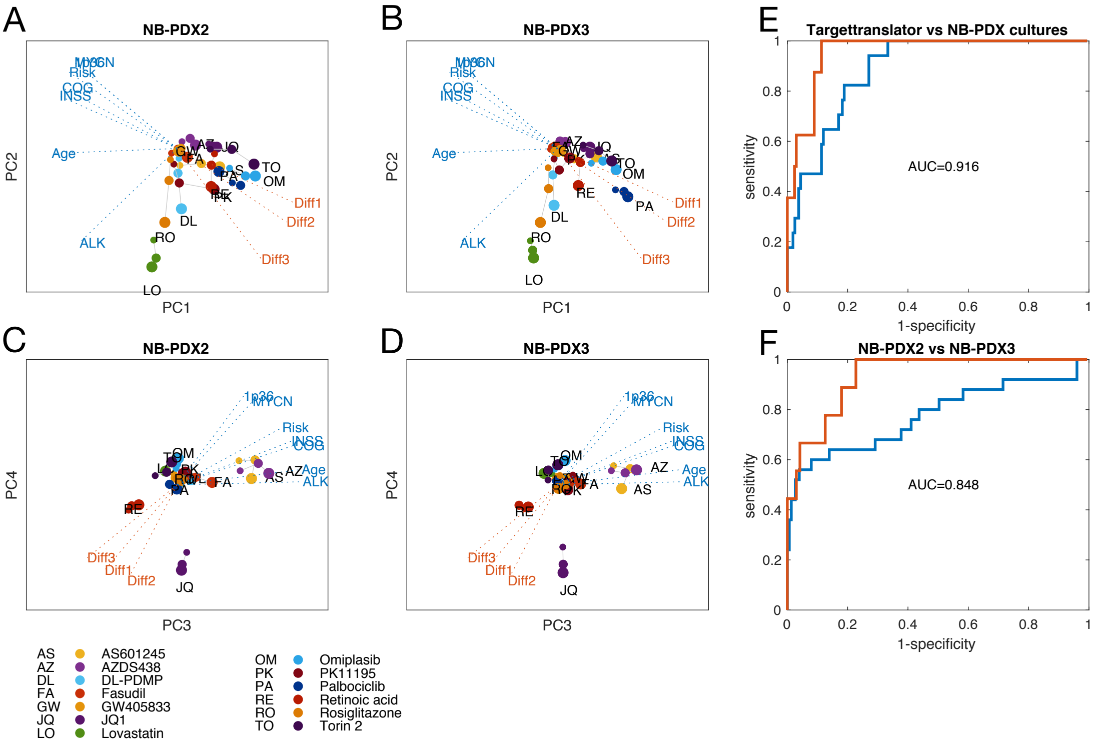

Supplementary Figure 3: 384-drugSeq validation of TargetTranslator prediction. (A-D) Comparison of 384-drugSeq results for the two neuroblastoma PDX cell cultures, NB-PDX2 and NB-PDX3. (E) ROC curves show agreement between our L1000-based predictions and the outcome in the PDX cultures. Red = classification of upregulation vs no effect, Blue = classification of downregulation vs no effect. (F) the agreement between the two different PDX cultures.

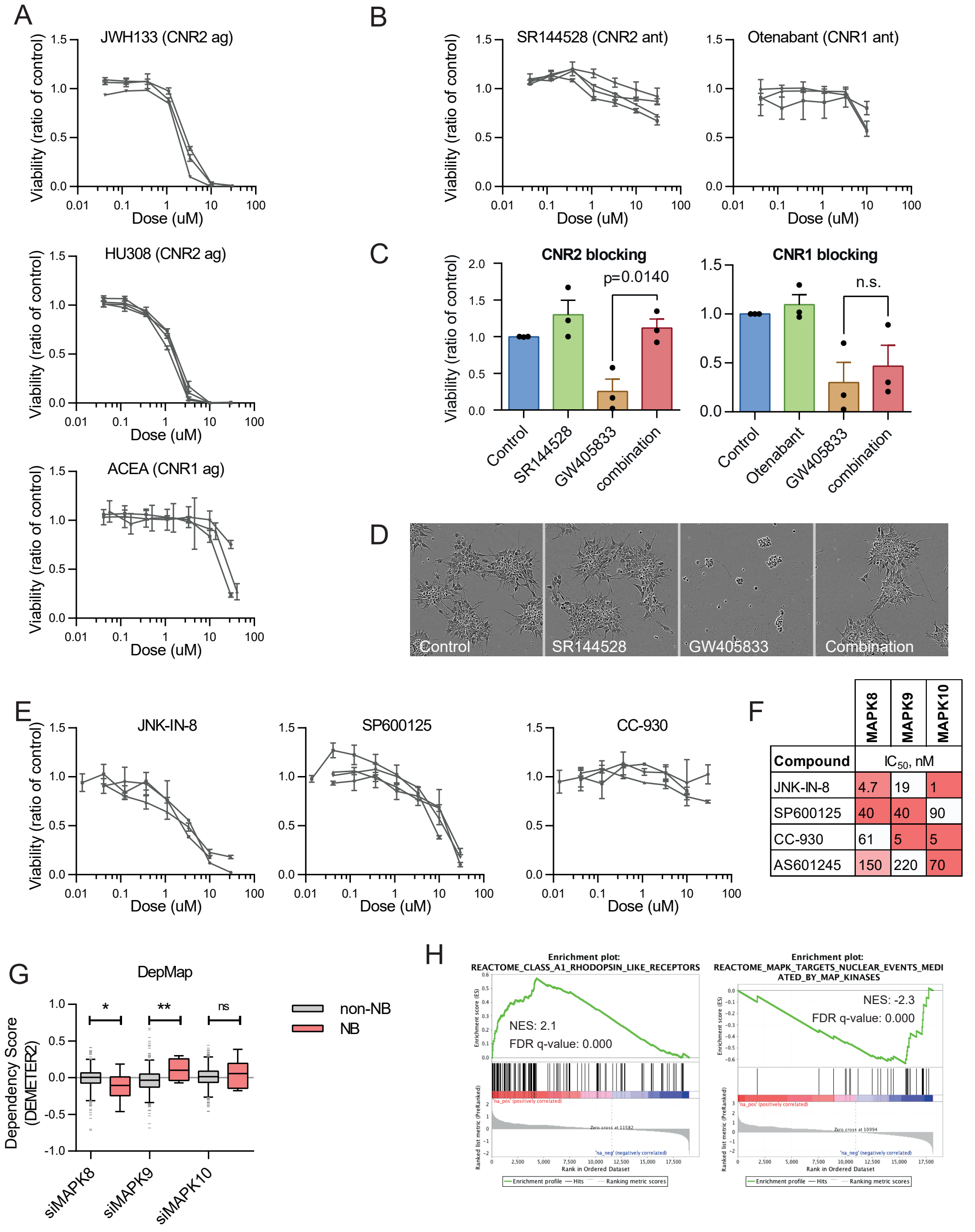

Compound	MAPK8	MAPK9	MAPK10
	IC$_{50}$, nM		
JNK-IN-8	4.7	19	1
SP600125	40	40	90
CC-930	61	5	5
AS601245	150	220	70

Supplementary Figure 4: Pharmacological selectivity of CNR1/2 and MAPK8/9/10 interventions in neuroblastoma cells. (A) CNR2 agonists JWH133 and HU308 inhibit neuroblastoma growth, while CNR1 agonist ACEA show limited effect. (B) Neither CNR1 nor CNR2 antagonist inhibited neuroblastoma growth. (C) Pre-treatment with CNR2 antagonist SR144528 significantly blocked the effect of GW405833, while CNR1 antagonist otenabant did not. (D) Representative images of SR144528 blocking of GW403833 viability decrease. (E) Viability of neuroblastoma cells after treatments of MAPK8/9/10 inhibitors JNK-IN-8, SP600125, and CC-930. (F) Isoform affinity for four MAPK8/9/10 inhibitors. red=higher affinity, white=lower affinity (G) DepMap dependency score (DEMETER2) for combined RNAi screen (Broad, Novartis, Marcotte) after knockdown of MAPK8/9/10 isoforms show a neuroblastoma (n=9) vulnerability for MAPK8 compared to all other cell lines (n=699). (H) GSEA enrichment after 6 h of treatments with GW405833 (left, top 1 positive enrichment score) or AS601245 (right, top 2 negative enrichment score) in NB-PDX2 and NB-PDX3.

Statistics: (A, B, E) mean, standard deviation, each line is an experimental replicate, (C) mean and SEM of three biological replicates, Student's t-test (G) 2.5-97.5% Box plot; Student's t-test with Bonferroni correction; $* = p < 0.05, ** = p < 0.001$.

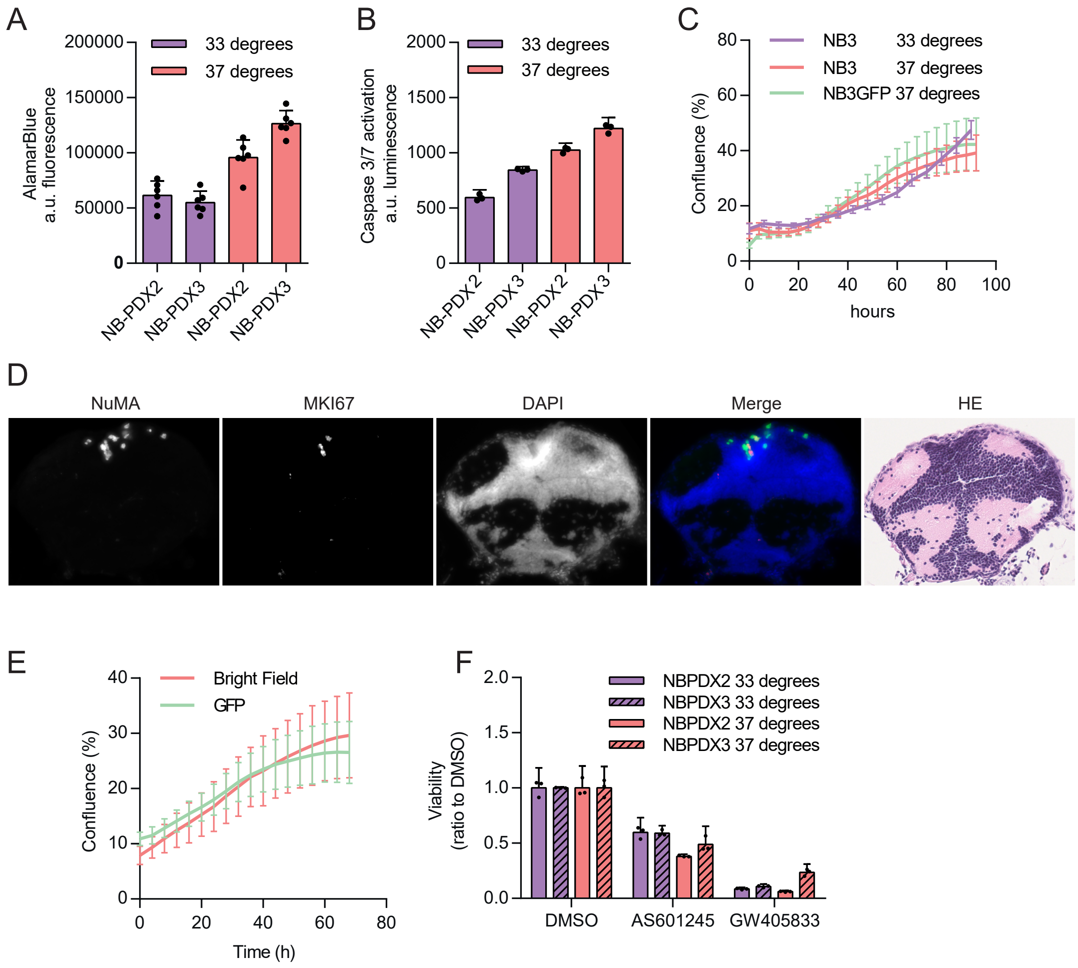

Supplementary Figure 5: Characterization of a zebrafish xenograft model of neuroblastoma. (A) Viability of neuroblastoma cells in 33 or 37 degrees C 72 h after seeding. (B) Apoptosis (cleaved caspase 3/7) of neuroblastoma cells in 33 or 37 degrees after 72 h. (C) Proliferation over 96 h of NB-PDX3 (used for zebrafish xenografts) in 33 or 37 degrees C, and for GFP-tagged NB-PDX3 in 37 degrees C. (D) Zebrafish xenografts contain proliferating (MKI67-positive) NB-PDX3 cells (human marker NuMA) at 5 dpf. (E) Confluence (%) based on automatic segmentation of bright field images or segmented GFP signal in the same cells over 72 h (GFP-tagged NB-PDX3 cells, n=15). (F) Effect of temperature on 72 h treatment with AS601245 and GW405833
Statistics: (A,B,D) mean, 95 % Confidence interval, (C,E) mean, standard deviation.

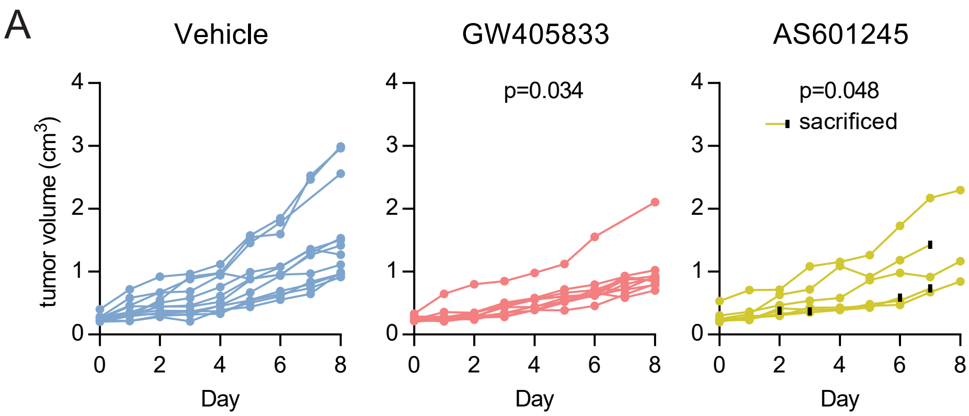

Supplementary Figure 6: Individual growth curves in mouse treatment study. (A) Tumor volume of all individual mice over 8 days of treatment. The AS601245 treatment group (n=8) was terminated due to unexpected toxicity. Mice that were sacrificed before study endpoint are marked as sacrificed.

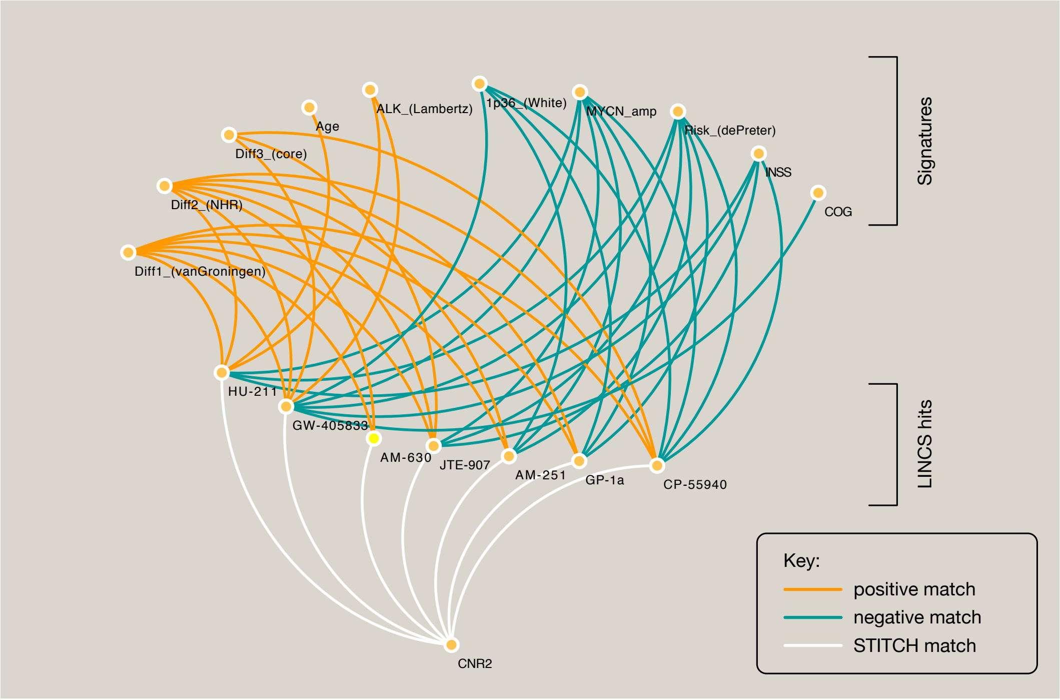

Supplementary Figure 7: CNR2-associated drugs with strong match scores. Visualization of figh ranking (TargetTranslator score > 0.2) drugs with a STITCH association (stitch score > 900). Generated using Cytoscape.

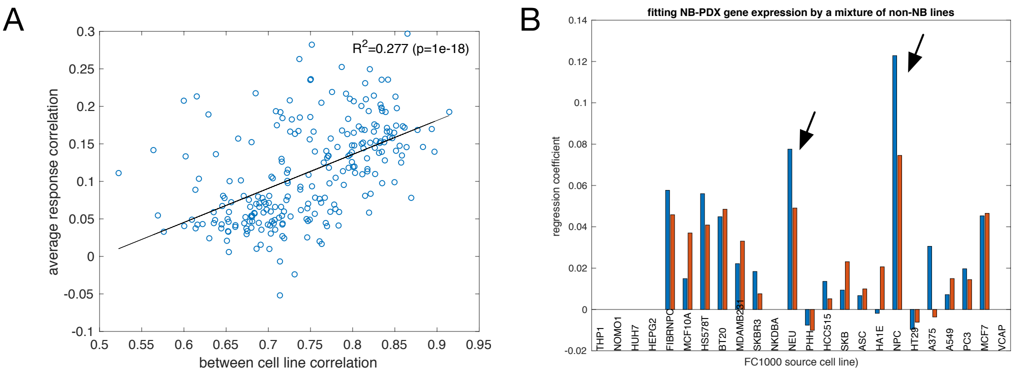

Supplementary Figure 8: Impact of cell lineage on correlations. (A) Analysis of the relation between the pairwise correlation of baseline gene expression between cell lines (x axis), and the average correlation of drugs induced transcriptional responses (log fold change) in the FC1000 data. Note that similar cell lines (before treatment) produce similar responses (after treatment). (B) Weights from a linear regression in which the log fold change observed in our NB-PDX2 and NB-PDX3 lines was modeled as a linear weighted function of the 24 cell lines in FC1000. The average regression weight for all 13 drugs in our RNAseq experiment is shown. Letter codes indicate L1000 cell line designations. Blue=weights for NB-PDX2 data, Red=weights for NB-PDX3 data. Arrows mark the top lines, NEU and NPC which are neuron and neural progenitor cultures, respectively.

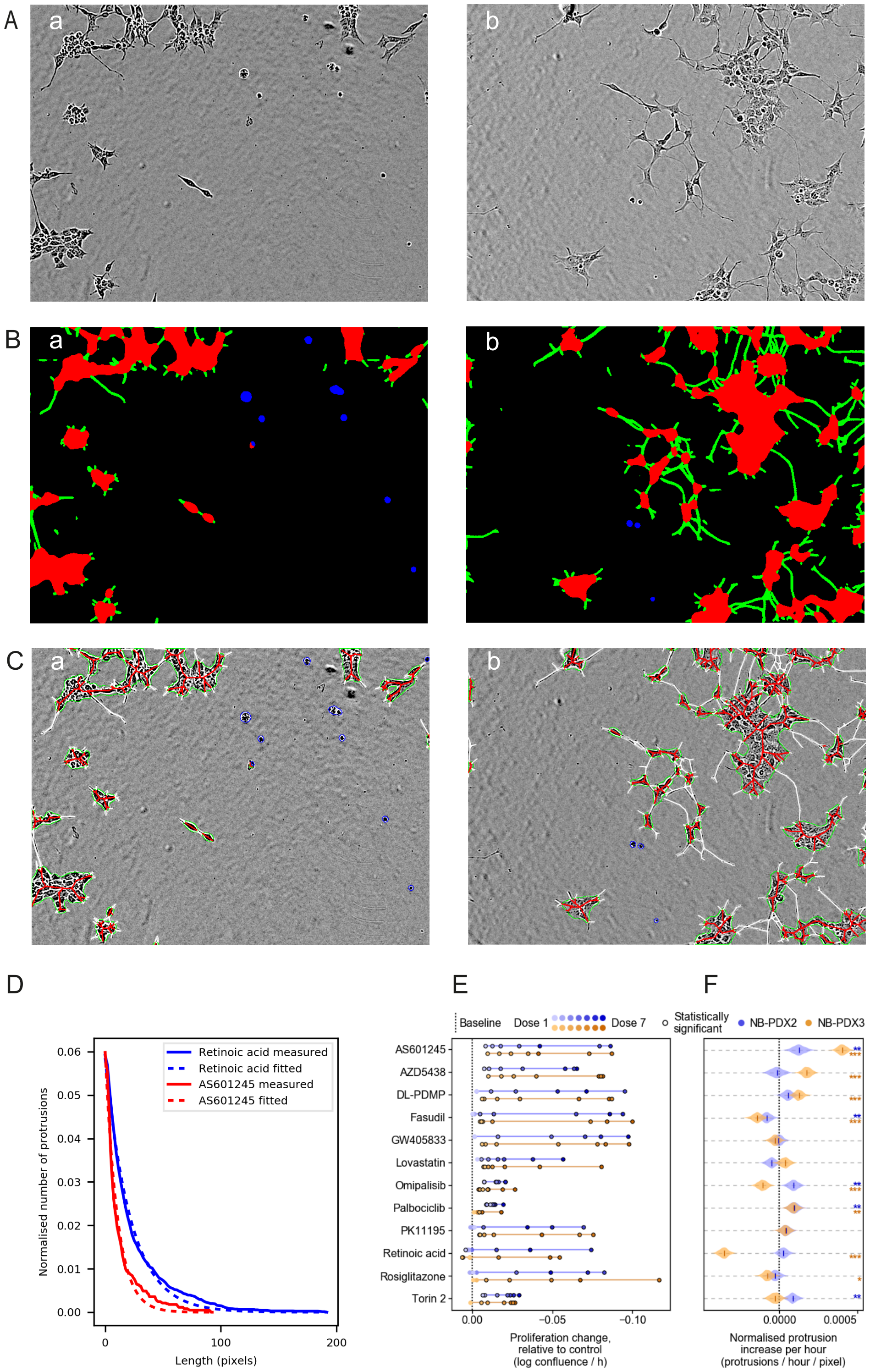

Supplementary Figure 9: Image analysis of adherent in vitro cells. (A - C) Example of image analysis for (a) AS601245 and (b) retinoic acid using IC10 dose after 72h of treatment on NB-PDX2. (A) Incucyte images. (B) Segmented images from CNN(red: cells, green: cell protrusion, blue: dead cells, black: background. (C) Overlay of skeletonization of cell protrusions over original images(white: cell protrusions, red: cell skeleton, green: cells, blue: dead cells). (D) Distribution of protrusion lengths from images in A. (E) Estimated compound dose effects on confluence for NB-PDX2 (blue) and NB-PDX3 (orange). Estimates with a black circle are statistically significant (q<0.05) compared with the baseline. (F) Bootstrapped (n=1000) estimates of change in number of protrusions, normalized by the length of the cell - circumference interface for NB-PDX2 (blue) and NB-PDX3 (orange). IC10 were used for all compounds. Stars show significance levels compared with negative control. $(* = p < 0.05, ** = p < 0.01, *** = p < 0.001)$

Supplementary Table 1: linear regression of principal components in Figure 4 with dose and time as co-variates. $* = p < 0.05, ** = p < 0.01, and *** = p < 0.001.$

	PC1		PC2		PC3		PC4	
	dose	time	dose	time	dose	time	dose	time
Control (water)								
Control (DMSO)								
AS601245	***	*						
AZD5438	**		*	***	***	*		***
DL-PDMP			***	**				*
Fasudil	**	*		*	***	**	*	**
GW-405833					*			
JQ1		***		**		***	***	
Lovastatin		**	***	***	**	*		**
Omipalisib	*			***		***	**	*
PK-11195	***		**		*	*		
Palbociclib		**		***	*	**		
Retinoic Acid		***	***	***		**		
Rosiglitazone	**	**	***	***			**	*
Torin-2	**			***		**	**	

PC1 vs dose, p<0.05, plots:

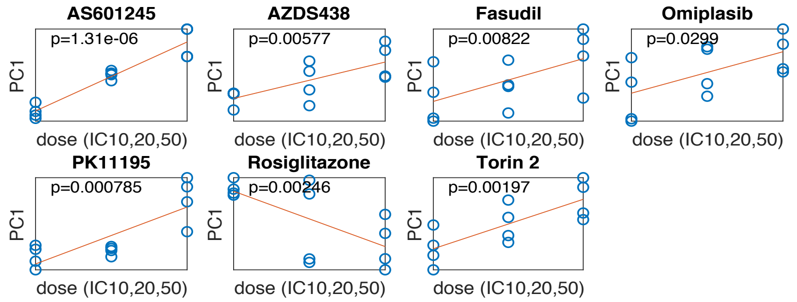

PC2 vs time, p<0.05, plots:

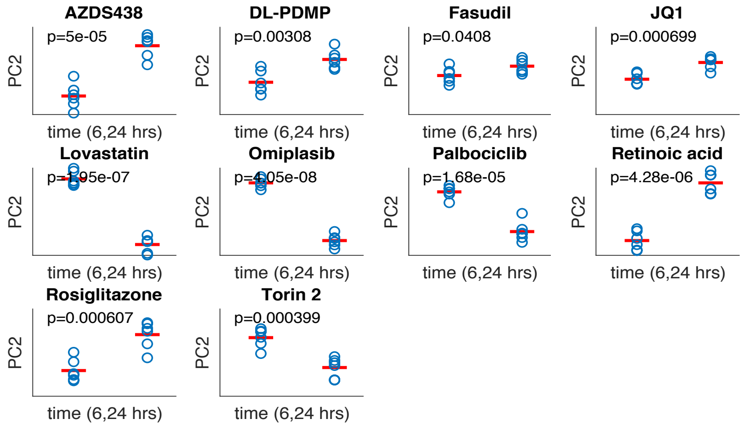

Supplementary Table 2: RNA-Seq dose table. Compound dose range used for RNA-Seq (μM). Dose 1, 2 and 3 correspond to IC10, IC20 and IC50 for each compound respectively.

Compound	Dose 3	Dose 2	Dose 1
AS601245	6	3	1.5
AZD5438	0.6	0.4	0.2
DL-PDMP	37	18.5	9.25
fasudil	40	20	10
GW405833	0.8	0.4	0.2
JQ1	1	0.5	0.25
lovastatin	6	3	1.5
omipalisib	0.05	0.025	0.0125
palbociclib	0.4	0.2	0.1
PK11195	70	35	17.5
retinoic acid	40	20	10
rosiglitazone	50	25	12.5
Torin 2	0.05	0.025	0.0125

Supplementary References

[1] Szklarczyk, D. *et al.* STITCH 5: augmenting protein-chemical interaction networks with tissue and affinity data. *Nucleic Acids Res.* **44**, D380–384 (2016).

[2] Molenaar, J. J. *et al.* Sequencing of neuroblastoma identifies chromothripsis and defects in neuritogenesis genes. *Nature* **483**, 589–593 (2012).

[3] Wang, C. *et al.* The concordance between RNA-seq and microarray data depends on chemical treatment and transcript abundance. *Nat. Biotechnol.* **32**, 926–932 (2014).

[4] Lonnstedt, I. M. & Nelander, S. FC1000: normalized gene expression changes of systematically perturbed human cells. *Stat Appl Genet Mol Biol* **16**, 217–242 (2017).

[5] Gagnon-Bartsch, J. A. & Speed, T. P. Using control genes to correct for unwanted variation in microarray data. *Biostatistics* **13**, 539–552 (2012).

[6] Chen, B. *et al.* Reversal of cancer gene expression correlates with drug efficacy and reveals therapeutic targets. *Nat Commun* **8**, 16022 (2017).

[7] Braekeveldt, N. *et al.* Neuroblastoma patient-derived orthotopic xenografts retain metastatic patterns and geno- and phenotypes of patient tumours. *Int. J. Cancer* **136**, E252–261 (2015).

[8] Persson, C. U. *et al.* Neuroblastoma patient-derived xenograft cells cultured in stem-cell promoting medium retain tumorigenic and metastatic capacities but differentiate in serum. *Sci Rep* **7**, 10274 (2017).

[9] Xie, Y. *et al.* The Human Glioblastoma Cell Culture Resource: Validated Cell Models Representing All Molecular Subtypes. *EBioMedicine* **2**, 1351–1363 (2015).

[10] Pollard, S. M. *et al.* Glioma stem cell lines expanded in adherent culture have tumor-specific phenotypes and are suitable for chemical and genetic screens. *Cell Stem Cell* **4**, 568–580 (2009).

[11] Picelli, S. *et al.* Full-length RNA-seq from single cells using Smart-seq2. *Nat Protoc* **9**, 171–181 (2014).

[12] Bolger, A. M., Lohse, M. & Usadel, B. Trimmomatic: a flexible trimmer for Illumina sequence data. *Bioinformatics* **30**, 2114–2120 (2014).

[13] Dobin, A. *et al.* STAR: ultrafast universal RNA-seq aligner. *Bioinformatics* **29**, 15–21 (2013).

[14] Anders, S., Pyl, P. T. & Huber, W. HTSeq–a Python framework to work with high-throughput sequencing data. *Bioinformatics* **31**, 166–169 (2015).

[15] Love, M. I., Huber, W. & Anders, S. Moderated estimation of fold change and dispersion for RNA-seq data with DESeq2. *Genome Biol.* **15**, 550 (2014).

[16] Subramanian, A. *et al.* Gene set enrichment analysis: a knowledge-based approach for interpreting genome-wide expression profiles. *Proc. Natl. Acad. Sci. U.S.A.* **102**, 15545–15550 (2005).

[17] Ronneberger, O., Fischer, P. & Brox, T. U-net: Convolutional networks for biomedical image segmentation. In Navab, N., Hornegger, J., Wells, W. M. & Frangi, A. F. (eds.) *Medical Image Computing and Computer-Assisted Intervention – MICCAI 2015*, 234–241 (Springer International Publishing, Cham, 2015).

[18] Chollet, F. Keras. `https://keras.io` (2015).

[19] Abadi, M. *et al.* TensorFlow: Large-scale machine learning on heterogeneous systems (2015). URL `https://www.tensorflow.org/`. Software available from tensorflow.org.

[20] Bates, D., Mächler, M., Bolker, B. & Walker, S. Fitting linear mixed-effects models using lme4. *Journal of Statistical Software* **67**, 1–48 (2015).

[21] White, R. M. *et al.* Transparent adult zebrafish as a tool for in vivo transplantation analysis. *Cell Stem Cell* **2**, 183–189 (2008).
