# Supplementary material for: Integrative discovery of treatments for high-risk neuroblastoma
Source: Nat Commun. 2020 Jan 3;11:71. doi: 10.1038/s41467-019-13817-8 (PMC6941971; doi:10.1038/s41467-019-13817-8)
Supplement: Supplementary file 3 — Description of Additional Supplementary Files [file 41467_2019_13817_MOESM3_ESM.pdf]

Description of Additional Supplementary Files

File Name: Supplementary Data 1
Description: Neuroblastoma gene signatures used for predictions
