# Supplementary material for: Integrative discovery of treatments for high-risk neuroblastoma
Source: Nat Commun. 2020 Jan 3;11:71. doi: 10.1038/s41467-019-13817-8 (PMC6941971; doi:10.1038/s41467-019-13817-8)
Supplement: Supplementary file 5 — Reporting Summary [file 41467_2019_13817_MOESM5_ESM.pdf]

nature research

Corresponding author(s): sven.nelander@igp.uu.se

Last updated by author(s): Nov 5, 2019

Reporting Summary

Nature Research wishes to improve the reproducibility of the work that we publish. This form provides structure for consistency and transparency in reporting. For further information on Nature Research policies, see Authors & Referees and the Editorial Policy Checklist.

Statistics

For all statistical analyses, confirm that the following items are present in the figure legend, table legend, main text, or Methods section.

n/a	Confirmed	
☐	☒	The exact sample size (*n*) for each experimental group/condition, given as a discrete number and unit of measurement
☐	☒	A statement on whether measurements were taken from distinct samples or whether the same sample was measured repeatedly
☐	☒	The statistical test(s) used AND whether they are one- or two-sided<br>*Only common tests should be described solely by name; describe more complex techniques in the Methods section.*
☐	☒	A description of all covariates tested
☐	☒	A description of any assumptions or corrections, such as tests of normality and adjustment for multiple comparisons
☐	☒	A full description of the statistical parameters including central tendency (e.g. means) or other basic estimates (e.g. regression coefficient) AND variation (e.g. standard deviation) or associated estimates of uncertainty (e.g. confidence intervals)
☐	☒	For null hypothesis testing, the test statistic (e.g. *F*, *t*, *r*) with confidence intervals, effect sizes, degrees of freedom and *P* value noted<br>*Give P values as exact values whenever suitable.*
☐	☒	For Bayesian analysis, information on the choice of priors and Markov chain Monte Carlo settings
☐	☒	For hierarchical and complex designs, identification of the appropriate level for tests and full reporting of outcomes
☐	☒	Estimates of effect sizes (e.g. Cohen's *d*, Pearson's *r*), indicating how they were calculated

Our web collection on statistics for biologists contains articles on many of the points above.

Software and code

Policy information about availability of computer code

Data collection	We parsed tab delimited text files from the R2 and TARGET consortia and the STITCH database (URLs in the Methods section), using perl scripts. L1000 data was prepared using methods described in Lönnstedt and Nelander (FC1000 R package, PMID: 28862994).
Data analysis	Data analysis was performed using a custom software, TargetTranslator. (source code at GitHub, tool at targettranslator.org)

For manuscripts utilizing custom algorithms or software that are central to the research but not yet described in published literature, software must be made available to editors/reviewers. We strongly encourage code deposition in a community repository (e.g. GitHub). See the Nature Research guidelines for submitting code & software for further information.

Data

Policy information about availability of data

All manuscripts must include a data availability statement. This statement should provide the following information, where applicable:

- Accession codes, unique identifiers, or web links for publicly available datasets
- A list of figures that have associated raw data
- A description of any restrictions on data availability

The sequencing data that support the findings of this study have been deposited in the Gene Expression Omnibus (GEO) with the accession code GSE120920 [https://www.ncbi.nlm.nih.gov/geo/query/acc.cgi?acc=GSE120920]. The source data underlying Figure 4G, 5, 6B, 6E, 7 and Supplementary Figures 4, 5, 6, 9, are provided in the Source Data file. Signatures used in the manuscript are defined in Supplementary Data 1. Additional data files, including the processed LINCS/L1000 and STITCH data can be accessed at targettranslator.org/downloads. For information on materials, contact SN (sven.nelander[at]igp.uu.se).

Field-specific reporting

Please select the one below that is the best fit for your research. If you are not sure, read the appropriate sections before making your selection.

☒ Life sciences ☐ Behavioural & social sciences ☐ Ecological, evolutionary & environmental sciences

For a reference copy of the document with all sections, see nature.com/documents/nr-reporting-summary-flat.pdf

Life sciences study design

All studies must disclose on these points even when the disclosure is negative.

Sample size	The sample size of the R2, TARGET, SEQ, L1000 and STITCH datasets used for integrative analysis is fixed. R2 contains 86 patients, TARGET contains 249 patients, and SEQ 498 patients.
Data exclusions	The RNA sequencing contained a QC step in which wells with low read count were removed (Methods).
Replication	The experiments contained technical and biological replicates (Methods).
Randomization	Randomization was applied for zebrafish and mice treatment studies.
Blinding	Blinding could not be done as it was the same researcher who treated and weighted the mice.

Reporting for specific materials, systems and methods

We require information from authors about some types of materials, experimental systems and methods used in many studies. Here, indicate whether each material, system or method listed is relevant to your study. If you are not sure if a list item applies to your research, read the appropriate section before selecting a response.

Materials & experimental systems

n/a	Involved in the study	
☐	☒	Antibodies
☐	☒	Eukaryotic cell lines
☒	☐	Palaeontology
☐	☒	Animals and other organisms
☒	☐	Human research participants
☒	☐	Clinical data

Methods

n/a	Involved in the study	
☒	☐	ChIP-seq
☒	☐	Flow cytometry
☒	☐	MRI-based neuroimaging

Antibodies

Antibodies used	1) Anti-n-MYC mouse monoclonal antibody (NCM II 100), Abcam, ab16898, lot: GR294469-1<br>2) Anti-cyclophilinB rabbit polyclonal antibody, Abcam, ab16045<br>3) Anti-NuMA rabbit polyclonal antibody, Abcam, ab97585<br>4) Anti-Cleaved PARP Rabbit monoclonal antibody, XP, Cell Signaling, #5625.
Validation	1) The antibody has been validated by the manufacturer and in house (data not shown) using cell lysate from all cell lines used in the manuscript and with 3013 and SK-N-SH as negative control.<br>2) The antibody has been validated by the manufacturer with knock-down experiments. The antibody also has 47 previous publications according to the manufacturer, lately in Nature Communications: The CPLANE protein Intu protects kidneys from ischemia-reperfusion injury by targeting STAT1 for degradation. Wang et al, 26 mars 2018.<br>3) The antibody has been validated by the manufacturer, has been extensively published, lately in Nature Medecine, PMID: 24076665. It showed no cross-reactivity with zebrafish cells.<br>4) The antibody has been extensively published, e.g. in PMID: 29029480, and has been validated by the manufacturer.

Eukaryotic cell lines

Policy information about cell lines

Cell line source(s)	Primary cell lines (NB and GBM) were provided by the original sources of cell line development (GBM: hgcc.org, PMID: 26629530 , NB: PMID: 25220031).<br>SK-N-BE(2) and SK-N-SH cell lines were acquired from ATCC (CRL-2271 and HTB-11 respectively).
Authentication	Primary cells were provided from the original source. Serum cultured cell lines has not been authenticated.

Mycoplasma contamination	Cells have been tested for mycoplasma infection. Positive cells have been discarded.
Commonly misidentified lines (See ICLAC register)	N/A

Animals and other organisms

Policy information about studies involving animals; ARRIVE guidelines recommended for reporting animal research

Laboratory animals	Wild type AB zebrafish (Danio rerio) embryos (up to 5 days) were used for the toxicity study and casper (PMID: 18371439) zebrafish embryos (up to five days) were used for the treatment study. For the mouse xenograft study, 5-6 weeks female nude mice (NMRI-nu/nu, Taconic) were used.
Wild animals	N/A
Field-collected samples	N/A
Ethics oversight	All zebrafish experiments have been approved by the regional ethics boards, Uppsala Djurförsöks etiska nämnd, ethical permits EP 161/14 and C68/15. All mouse experiments were approved by the regional ethics committee for animal research (N 231/14), appointed and under the control of the Swedish Board of Agriculture and the Swedish Court.

Note that full information on the approval of the study protocol must also be provided in the manuscript.
